# A Rare Complication of Trimethoprim-Sulfamethoxazole: Drug Induced Aseptic Meningitis

**DOI:** 10.1155/2016/3879406

**Published:** 2016-08-07

**Authors:** Pinky Jha, Jeremiah Stromich, Mallory Cohen, Jane Njeri Wainaina

**Affiliations:** ^1^Section of Hospital Medicine, Division of General Internal Medicine, Medical College of Wisconsin, 9200 West Wisconsin Avenue, Milwaukee, WI 53226, USA; ^2^Medical College of Wisconsin, 9200 West Wisconsin Avenue, Milwaukee, WI 53226, USA; ^3^Children's Hospital of Wisconsin, 9200 West Wisconsin Avenue, Milwaukee, WI 53226, USA; ^4^Section of Perioperative Medicine and Division of Infectious Diseases, Department of Medicine, Medical College of Wisconsin, 9200 West Wisconsin Avenue, Milwaukee, WI 53226, USA

## Abstract

Drug induced aseptic meningitis is a rare but challenging diagnosis, most commonly reported with nonsteroidal anti-inflammatory drugs and antibiotics. Trimethoprim/sulfamethoxazole is a sulfonamide that is widely used in clinical practice for the treatment and prophylaxis of various infections. Drug induced aseptic meningitis, when seen with trimethoprim/sulfamethoxazole, occurs predominantly in patients with some degree of immune compromise and is less commonly seen in immune competent individuals. The patient often exhibits the classic symptoms of meningitis. Early diagnosis is important, since the cessation of the antibiotic leads to rapid clinical improvement. Trimethoprim/sulfamethoxazole induced aseptic meningitis has been underreported to FDA/MED-WATCH program. Here we report two cases of trimethoprim/sulfamethoxazole: an immune competent individual and immune compromised individual, both of which presented with signs of meningitis and a negative infectious workup. Trimethoprim/sulfamethoxazole is an uncommon and mysterious adverse reaction to a commonly used antibiotic. It should be considered in the differential diagnosis of patients presenting with acute signs and symptoms of meningitis especially after infectious causes have been ruled out.

## 1. Introduction

Meningitis is an inflammatory condition of the meninges that is usually caused by a viral or bacterial infection. It is important to differentiate the etiology of meningitis into aseptic and bacterial because of the differences in severity of illness, treatment modalities, and prognostic implications. Aseptic meningitis refers to meningeal inflammation that is not caused by bacterial infection as evidenced by negative bacterial cerebrospinal fluid (CSF) culture. Drugs, viruses, and malignancies are known causes of aseptic meningitis. Drug induced aseptic meningitis (DIAM) is an uncommon and mysterious adverse reaction to certain medications, most commonly NSAIDs and antibiotics. Trimethoprim/sulfamethoxazole (TMP-SMX) is an antibiotic combination that is widely used for prophylaxis and treatment of infections. TMP-SMX has excellent tissue penetration into, for example, bone, prostate, and the central nervous system (CNS). Rare but sometimes fatal reactions have occurred with use of sulfonamides. These reactions, among many others, include aseptic meningitis. DIAM seen with TMP-SMX occurs predominantly in patients with some degree of immune compromise resulting from HIV/AIDS, organ transplantation, collagen vascular disease, and malignancy. This is logical as these individuals are much more likely to require TMP-SMX for prophylaxis or treatment as compared to young immunocompetent individuals where DIAM from TMP-SMX is rarely seen. Here we report two cases of TMP-SMX induced aseptic meningitis (TSIAM) in immune compromised and immune competent hosts, respectively.

## 2. Case 1

A 26-year-old female with no significant past medical history presented to the emergency department (ED) with a severe headache and fever for 2 days. The pain was located at the top of her head and radiated to her neck. On exam she was in severe distress due to the headache, was tachycardiac, and had a fever of 101.1°F. She had nuchal rigidity and Kernig's sign was positive. The rest of the physical exam was normal. Laboratory data revealed a white blood cell count (WBC) of 6000/cm^3^, hematocrit of 38.8%, and normal blood chemistry. Urine toxicology screening and urinalysis and urine culture were negative. A chest X-ray was normal, the electrocardiogram showed sinus tachycardia, and a noncontrast computed tomography scan of the head was normal. She underwent a lumbar puncture and was started on empiric intravenous (IV) vancomycin, ceftriaxone, and acyclovir for possible bacterial meningitis based on the history and exam. Cerebrospinal fluid (CSF) analysis showed glucose 51 mg/dL, protein 110 mg/dL, WBC 152/*μ*L, red blood cell count 2/*μ*L, monocytes 16%, and polysegmented neutrophils 28%. CSF gram stain showed no polymorphonuclear cells (PMNs), few mononuclear cells, and no bacteria. CSF culture and Herpes Simplex virus polymerase chain reaction (PCR) were negative. Infectious disease (ID) consultation was requested. On further questioning she gave a history of using TMP-SMX for a UTI prior to admission. After completing the three-day course of TMP-SMX she had developed back pain followed by severe headache and fever. Given her clinical presentation, lack of evidence of an infectious etiology on CSF studies, and the temporal relationship with TMP-SMX use, a diagnosis of aseptic meningitis was made. IV antibiotics were stopped per ID recommendations and her symptoms completely resolved with supportive care. She was discharged to home in stable condition after three days and was advised to avoid TMP-SMX in the future.

## 3. Case 2

A 48-year-old male with a 20-year history of HIV infection presented to our ED with a headache and neck stiffness for five days. The patient also reported fever, photophobia, diffuse myalgias, and arthralgias. He denied vision changes, ataxia, aphasia, cough, diarrhea, sick contacts, and recent travel. The patient had been restarted on an antiretroviral regimen (Etravirine 200 mg BID, Raltegravir 400 mg BID, Ritonavir 100 mg BID, and Darunavir 600 mg BID) four weeks earlier after a year's hiatus. Prophylactic TMP-SMX double strength (800 mg/160 mg) one tab daily and azithromycin 12,000 mg every week had also been initiated at that time. His most recent HIV-1 viral load was 80,000 copies/mL and CD4 cell count was 58/*μ*L. Upon presentation, the patient was in severe distress due to the headache and photophobia. Temperature upon admission was 100.1°F with a heart rate of 90/min. On physical exam, the patient had nuchal rigidity along with positive Kernig's and Brudzinski's signs. The rest of the physical exam was unremarkable.

CSF analysis showed WBC of 11/*μ*L, with lymphocytes 49%, monocytes 28%, and PMNs 16%. Glucose was 60 mg/dL, protein was 79 mg/dL, and gram stain was negative. CT scan and MRI of the brain were normal. Cryptococcal antigen in CSF, Toxoplasma PCR in CSF and IgM/IgG in blood, CMV NAAT in CSF and blood, extended respiratory viral panel NAAT via nasal swab (consisting of Influenza A and Influenza B, Parainfluenza 1–3, RSV, Human Metapneumovirus, Rhinovirus, and Adenovirus), JC virus PCR in CSF, and Enterovirus NAAT in CSF were done to rule out other infections in this immune compromised patient and returned negative. All other laboratory studies were negative, including blood cultures. The patient was empirically started on IV acyclovir, ceftriaxone, and vancomycin in the ED but was switched from ceftriaxone to cefepime as per ID recommendation. He also required multiple doses of intravenous hydromorphone for his headache. By day three of hospitalization, IV acyclovir, cefepime, and vancomycin had been discontinued. Based on clinical presentation and laboratory results that spoke against bacterial meningitis, a diagnosis of aseptic meningitis was made. As there was no evidence for an infectious cause, TMP-SMX was stopped on day four of hospitalization given concern for a possible DIAM and the recent addition of TMP-SMX by his HIV provider.

Patient reported improvement in photophobia, headache, and neck stiffness the following day. He was discharged on day five of hospitalization. Upon followup a week after discharge, the patient reported complete resolution of symptoms.

## 4. Discussion

The two patients presented here were on TMP-SMX for treatment of a UTI and PCP prophylaxis, respectively. TMP-SMX induced aseptic meningitis is a rare but important and often challenging diagnosis.

The TMP-SMX combination was approved by US Food and Drug Administration in 1973. It was approved as a fixed-drug combination given the synergistic effect of the 2 medications producing sequential blockade of microbial dihydrofolate reductase. Each drug is widely distributed in the body and can be detected in most tissues including the CSF. The serum half-life of TMP is 8–10 hours and that of SMX is 10 hours [1]. TMP-SMX is widely used in clinical practice owing to its low cost and effectiveness in treating common infections such as UTI, traveler's diarrhea, and methicillin resistant* Staphylococcus aureus* skin and soft tissue infections. Use of this antibiotic for treatment and prophylaxis of* Pneumocystis jiroveci* pneumonia (PJP) has increased with the rise of HIV infection. It carries the additional advantage of providing protection against* Toxoplasma gondii* infection and reactivation, bacterial pneumonia, UTIs, and* Nocardia*,* Legionella*, and* Listeria* infections. Most commonly reported side effects from TMP-SMX are gastrointestinal upset and skin rashes [2].

The first documented case of DIAM was reported by Widener and Littman in 1978 in a young lady with systemic lupus on ibuprofen therapy [3]. Kremer et al. in 1983 reported a case of DIAM in a previously healthy female who developed headache, neck pain, nausea, and vomiting 3 hours after taking her first dose of TMP-SMX [4]. The most frequently encountered classes of medications involved in DIAM include NSAIDs, antibiotics (with TMP-SMX, the most frequently reported), antiepileptic drugs, and immunosuppressive/immunomodulatory agents (Table 1). Smilack reviewed forty-one reported cases of TSIAM and found a predominance of female patients and patients with autoimmune disease. Six out of forty-one cases were on TMP-SMX for PJP prophylaxis [1].

The frequent use of prophylactic TMP-SMX in HIV infected patients increases the probability of TSIAM in this population [6]. This clinical diagnosis should be included in the list of diagnostic possibilities considered in persons infected with HIV presenting with symptoms of meningitis. Meningitis due to* Cryptococcus*, coccidioidomycosis, histoplasmosis, or other fungal infection represents an AIDS-defining event and occurs typically with very low CD4 counts [7]. The patient discussed here had a CD4 count of 58/*μ*L, which made a fungal etiology such as those listed above possible but all infectious etiologies were ruled out during his workup prior to the conclusion that he had TSIAM.

TSIAM has been well reported in the literature with over 33 case reports and 41 documented patients [5]. However, the incidence and pathogenic mechanism of this disease are still uncertain. The incidence remains unknown as most cases are not reported and many remain unrecognized [7]. It does not appear to be dose or time related. The mechanism of reaction does not fit into the typical Gell and Coombs classification, although it likely represents an immune-mediated hypersensitivity reaction. It has been observed that shorter intervals between ingestion and symptom onset occur on repeated exposures which supports an allergic mechanism [2]. An IgE mediated response is less likely in absence of typical finding of urticaria, angioedema, or bronchospasm and due to the lack of an immediate response [8]. Possible mechanisms therefore include a nonimmediate hypersensitivity reaction, direct drug toxicity, and immune complex deposition [9]. Antonen et al. [10] suggested IL-6 as a possible mediator.

Symptoms of DIAM usually include mild headache, low grade fever, photophobia, and neck stiffness, although hemodynamic compromise and respiratory failure requiring intubation and admission to the intensive care unit have been reported [11]. The described symptoms can occur in patient naïve to TMP-SMX as well as in previous users. These symptoms can occur hours to weeks following administration of the drug. CSF analysis suggestive of TMP-SMX induced meningitis includes a neutrophilic pleocytosis and mildly elevated protein along with normal glucose and negative gram stain. Imaging of the brain is normal in most case reports [11]. Patients typically improve within 2-3 days of stopping the antibiotic, which corresponds to between 5 and 7 half-lives of the drug, at which point 95 to 99% of the medication has been eliminated. Treatment consists of withdrawing the medication and supportive care. However, as the symptoms and laboratory results are indistinguishable from partially treated bacterial meningitis, most patients with symptoms of meningitis are treated with intravenous antibiotics of another class and antivirals while awaiting bacterial and viral study results. Complete resolution of symptoms without relapse after stopping antibiotics rules out partially treated meningitis [8].

Drug induced aseptic meningitis is therefore a diagnosis of exclusion, made after infectious causes have been ruled out. A temporal relationship between the use of the drug and subsequent onset of meningeal symptoms, negative CSF culture, and resolution of symptoms after drug withdrawal are the keys to diagnosis. It is important to note that symptoms may take days to develop after the first exposure to the drug. In fact, some patients initially tolerate the offending drug without complications. Repeated exposures to the drug, however, will always demonstrate a quicker onset of symptoms, usually within hours. Thus, a definitive diagnosis can be made, if necessary, with a drug rechallenge in a controlled setting [11].

Recognition and diagnosis of TMP-SMX induced meningitis is important, as it is treatable by withdrawal of the drug and recurrence is thus prevented. The outcome is generally good, usually without long term sequelae. Recovery is complete most of the time within 3 to 11 days, even in the case of most severe symptoms [11].

## 5. Conclusion

There should be a high index of suspicion of DIAM in all patients presenting with symptoms of meningitis and negative CSF culture. A careful medication history including the temporal relationship between drug use and onset of symptoms must be obtained. Neutrophilic pleocytosis and negative cultures are essential for the diagnosis. The symptoms usually resolve quickly once the offending agent has been removed. Further research is needed to identify the mechanism of TSIAM and to identify factors that increase the risk for the reaction.

## Figures and Tables

**Table 1:** Drug induced aseptic meningitis.

Class and most frequently reported	# of cases^a^
NSAIDs	
Ibuprofen	42
Sulindac	7
Naproxen	7

Antibiotics	
TMP-SMX	32
TMP	11
Amoxicillin	8
Metronidazole	3

Immunosuppressive/immunomodulatory	
Cetuximab	5
Infliximab	4
Sulfasalazine	3

Antiepileptic	
Lamotrigine	30
Carbamazepine	4

^a^Adapted from Morís and Garcia-Monco 2014 [5].
